# A Defect in Lipoprotein Modification by Lgt Leads to Abnormal Morphology and Cell Death in Escherichia coli That Is Independent of Major Lipoprotein Lpp

**DOI:** 10.1128/jb.00164-22

**Published:** 2022-08-08

**Authors:** S. Legood, D. Seng, I. G. Boneca, N. Buddelmeijer

**Affiliations:** a Institut Pasteur, Université de Paris, CNRS UMR6047, INSERM U1306, Unité de Biologie et Génétique de la paroi bactérienne, Paris, France; University of Florida

**Keywords:** Lgt, viability, Lpp, cell envelope, *Escherichia coli*, cell viability

## Abstract

Lgt is an essential enzyme in proteobacteria and therefore a potential target for novel antibiotics. The effect of Lgt depletion on growth, morphology, and viability was studied in Escherichia coli to assess whether absence of Lgt leads to cell death. Two Lgt depletion strains were used in which *lgt* was under the control of an arabinose-inducible promoter that allowed regulation of Lgt protein levels. Reduced levels of Lgt led to severe growth and morphological defects that could be restored by expressing *lgt* in *trans*, demonstrating that only Lgt is responsible for the distorted phenotypes. In the absence of major lipoprotein Lpp, growth defects were partially restored when low levels of Lgt were still present; however, *lgt* could not be deleted in the absence of Lpp. Our results demonstrate that Lpp is not the main cause of cell death under conditions of Lgt depletion and that other lipoproteins are important in cell envelope biogenesis and cell viability. Specific inhibitors of Lgt are thus promising for the development of novel antibiotics.

**IMPORTANCE** Incomplete maturation and envelope mislocalization of lipoproteins, through inhibition or mutations in lipoprotein modification enzymes or transport to the outer membrane, are lethal in proteobacteria. Resistance to small-molecule inhibition or the appearance of suppressor mutations is often directly correlated with the presence of abundant outer membrane lipoprotein Lpp. Our results show that Lgt, the first enzyme of the lipoprotein modification pathway, is still required for growth and viability in the absence of Lpp and thus is necessary for the function of other essential lipoproteins in the cell envelope. This adds credence to the hypothesis that Lgt is essential in proteobacteria and an attractive target for the development of novel antibiotics.

## INTRODUCTION

Lipoproteins play an important role in physiology and viability of bacteria and are involved in essential processes for the cell envelope, such as cell wall biogenesis, transport and insertion of membrane proteins and lipopolysaccharides, nutrient uptake, and efflux of toxic molecules (1–3). They are anchored in membranes through their fatty acid-linked amino termini, which also play an important role in virulence by signaling the innate immune response via interaction with Toll-like receptors (4). Lipoprotein maturation is a posttranslational process that takes place in the cytoplasmic membrane and involves three essential integral membrane proteins in proteobacteria. Lipoproteins are synthesized in the cytoplasm with a Sec or Tat membrane-targeting sequence composed of a hydrophobic signal peptide containing a specific sequence, the so-called lipobox, for recognition by the lipoprotein modification machinery. The first step in the pathway is catalyzed by phosphatidylglycerol:prolipoprotein diacylglyceryl transferase (Lgt), which transfers a diacylglyceryl moiety from the phospholipid phosphatidylglycerol to the sulfur group of the lipoprotein-specific cysteine residue of prolipoprotein, resulting in a thioether-linked diacylglyceryl-cysteine and glycerol-1-phosphate by-product (5, 6). The second step involves cleavage of the signal peptide catalyzed by signal peptidase II (Lsp), liberating the α-amine group of diacylglyceryl-cysteine (7). The third and final step in the pathway is catalyzed by apolipoprotein N-acyltransferase, which adds a third fatty acid from the *sn*-1 acyl group of phosphatidylethanolamine onto the α-amine, resulting in mature triacylated lipoprotein (8, 9). Lipoprotein modification is key for the biogenesis and maintenance of the cell envelope. In Escherichia coli, more than 90 lipoproteins, of which at least 80 are located in the outer membrane, have been confirmed by fatty acid acylation (10), and three outer membrane lipoproteins have been reported as essential, i.e., the lipoprotein outer membrane receptor LolB (11), the lipopolysaccharide (LPS) assembly component LptE (12, 13), and the β-barrel assembly complex component BamD (14, 15). Major outer membrane lipoprotein Lpp (Braun’s lipoprotein) is not essential but is required for maintenance of the cell envelope. This highly abundant lipoprotein, with approximately 1 million copies per cell, is partly covalently cross-linked to the peptidoglycan (16) and surface exposed (17). Mislocalization of Lpp to the cytoplasmic membrane while being cross-linked to the peptidoglycan is lethal for E. coli (18).

Lgt catalyzes the first irreversible step in the sequential pathway of lipoprotein modification and is conserved in all bacteria (1). To better understand its role in bacterial physiology, we addressed its essentiality for viability of E. coli. Previous studies demonstrated that in conditions of incomplete maturation of lipoproteins, either by mutations (19) or upon inhibition of the modification enzymes by small molecules (20–23), mutations occur in *lpp* that lead to absence of the protein or the inability to cross-link to the peptidoglycan (20, 24). In this study, we showed that Lgt is essential in the absence of Lpp and that other lipoproteins play an important role in cell envelope biogenesis in E. coli, validating Lgt as a potential target for the development of novel antibiotics.

## RESULTS

### Lgt is essential for viability of E. coli.

**FIG 1:**
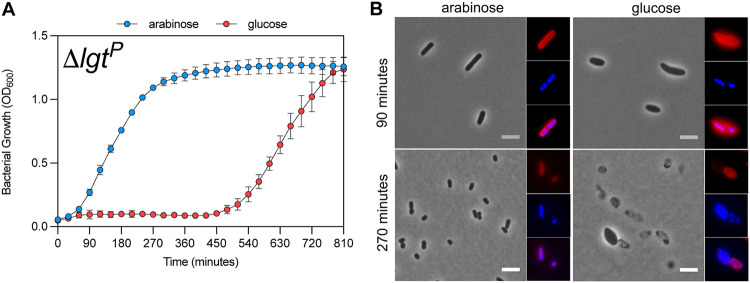
Lgt is essential for viability of E. coli. (A) Growth curve of *lgt* depletion strain (PAP9403, ΔlgtP) in 0.2% l-arabinose and 0.2% d-glucose. Time (t) 0 corresponds to 2 h of growth in LB medium without sugar. Graphed data represent duplicate OD_600_ measurements of biological triplicates. (B) Phase-contrast images of *lgt* depletion strain Δ*lgtP* grown in the presence of 0.2% l-arabinose and 0.2% d-glucose. Nucleoids were stained with Hoechst (blue), and the membrane was stained with FM4-64X (red). Bar, 5 μm.

To address the role of major lipoprotein Lpp on the essentiality of Lgt and to confirm previous reports that showed a decrease in cell viability when *lgt* expression was reduced, we employed the reported E. coli Lgt depletion strain PAP9403, in which the chromosomal *lgt* gene is replaced by a kanamycin resistance cassette in a manner that does not disrupt the downstream *thyA* gene (25). To sustain growth, the *lgt* gene is present on a plasmid (pBAD18s-Cm-*lgt*^WT^-c-*myc*_2_, pLgt) under the control of an arabinose-inducible promoter (P_ara_) (Δ*lgt*^P^). When PAP9403 (Δ*lgt*^P^) is grown in the absence of l-arabinose or in the presence of d-glucose, an initial phase of growth is observed for 90 min (5, 25), before the cultures exhibit a decrease in optical density suggesting growth arrest up to 450 min (about 20 generations) (Fig. 1). Upon depletion of Lgt, cells became wider, rounded up, lost DNA, displayed a membrane defect, and finally lysed (Fig. 1). These results confirmed that *lgt* is an essential gene in E. coli.

### Revertants of plasmid-encoded Lgt depletion are not affected in Lpp modification.

**FIG 2:**
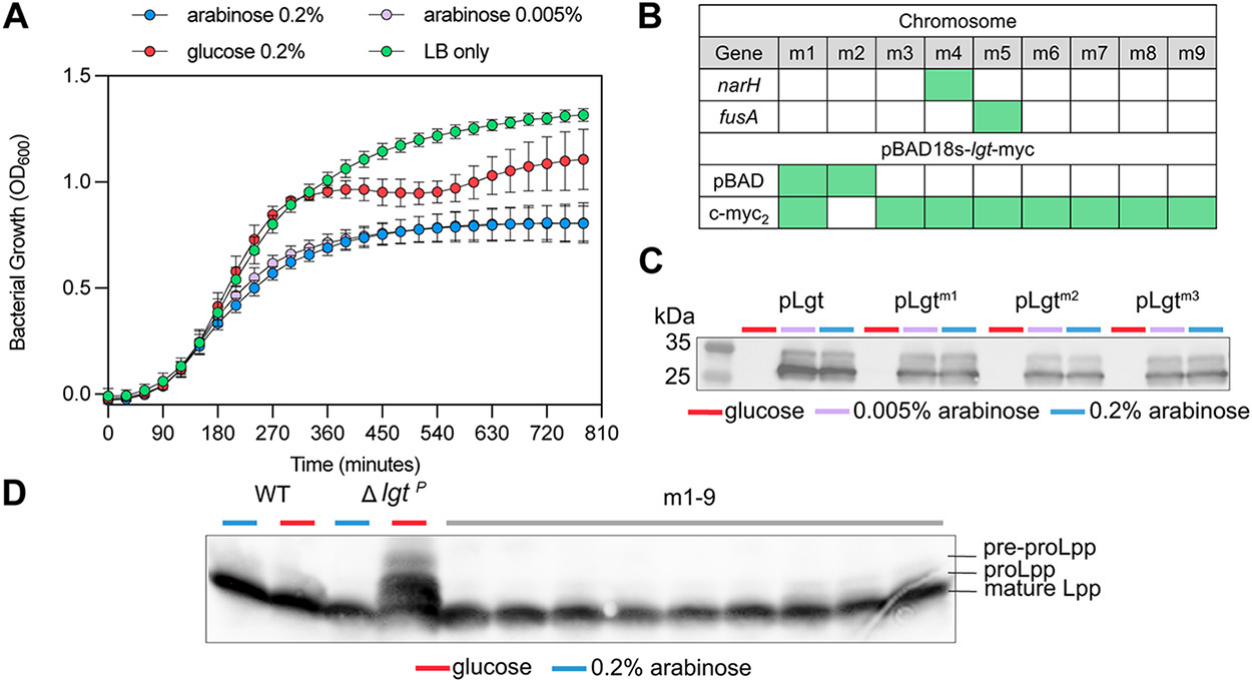
Mature form of Lpp is detected in revertants of the Lgt depletion strain. (A) Growth curve of revertants of strain PAP9403 (Δ*lgt*^P^) obtained after extensive growth under restrictive conditions. Grouped growth curves of nine revertants in the presence of high (0.2%) or low (0.005%) l-arabinose, 0.2% d-glucose, or in LB medium without sugar, are presented. (B) Chromosomal and plasmid mutations identified by WGS in 9 revertant strains (Δ*lgt*^Pm1^ to Δ*lgt*^Pm9^) are indicated as green boxes. In *narH* adenosine replaced thymine at position 322, resulting in Y108N in the protein, and *fusA* has thymine 1961 rather than adenosine, leading to I654N. For further details of pLgt mutations, see Fig. S1 in the supplemental material. (C) Detection of Lgt-*myc*_2_ on Western blotting by anti-c-*myc* antibodies (Sigma) from wild-type strain BW25113 retransformed with plasmids from revertants m1 to m3, representing all combinations of mutations. Strains were grown in the presence of high (0.2%) or low (0.005%) l-arabinose, or 0.2% d-glucose. (D) Detection of Lpp by Western blotting using anti-Lpp antibodies. An equal amount of total cell extract (0.1 OD_600_ units) from cultures grown for 270 min was loaded per lane.

We observed restored growth in the Lgt depletion strain under restrictive growth conditions at 450 min. We hypothesized that growth restoration occurred due to the absence of, or mutations in, Lpp that affected its interaction with the peptidoglycan. The arabinose-independent phenotype was maintained in all revertants from Δ*lgt*^P^ (m1 to -9), suggesting that the phenotype was passed on to future generations (Fig. 2). We performed whole-genome sequencing (WGS) to investigate mutations in *lpp* or other loci that could explain the revertant phenotype. Few single-nucleotide polymorphisms (SNPs) were identified in some of the revertants, either on the chromosome or on the pLgt plasmid (Fig. 2; see also Fig. S1 in the supplemental material). The mutations in *narH* and *fusA*, encoding nitrate reductase A subunit β and elongation factor G, respectively, were not directly involved in lipoprotein modification. No mutations were observed in *lpp* or *micL*, which encodes a small RNA regulating *lpp* (26), or in genes related to cell envelope biogenesis or the stress response. Strikingly, some revertants did not seem to have chromosomal mutations. Mutations were identified in pLgt, i.e., two revertants had an SNP in the arabinose promoter and eight had mutations in the 3′-end of the gene encoding the c-*myc* tag (see Fig. S1). This raised the possibility that mutations in the promoter of pLgt cause *lgt* expression independent of arabinose, resulting in Lgt production and therefore restoration of growth. pLgt from three revertants was isolated and transformed into wild-type strain BW25113, and clones were analyzed for Lgt production upon induction with l-arabinose and in the presence of d-glucose to repress expression of *lgt* from P_ara_ (Fig. 2). Whereas pLgt^m1^ had mutations in both the promoter region and the c-*myc* tag, pLgt^m2^ had a mutation in the arabinose promoter and pLgt^m3^ to pLgt^m9^ contained a mutation in the c-*myc* tag (see Fig. S1). Western blot analysis of Lgt-c-*myc*_2_ revealed slightly reduced levels of Lgt from the revertant plasmids compared to the original plasmid in the presence of l-arabinose. Lgt could not be detected in any of the strains in the presence of d-glucose, possibly due to detection limits of the Western blot assay. To test whether the revertant plasmids are required for restored growth, we used the pFREE system (27). This system is based on the elimination of plasmids through expression of CRISPR-Cas9 that targets the origin of replication region in plasmids. The nine plasmids from the revertant strains could not be lost, whereas pLgt was removed from a wild-type strain (see Fig. S2). This suggests that the plasmid-linked mutations lead to arabinose-independent low-level expression of *lgt-myc_2_*. Migration of Lpp on high-resolution gel electrophoresis revealed that all strains contained mature, triacylated Lpp, in contrast to Δ*lgt*^P^ grown with d-glucose, where accumulation of proLpp and pre-proLpp was observed (Fig. 2). This showed that maturation of Lpp was not affected in the revertants. Overall, the data suggested that very low levels of Lgt are sufficient to sustain growth, possibly in addition to an unidentified modification.

### Deletion of *lpp* partially restores growth of an Lgt depletion strain.

**FIG 3:**
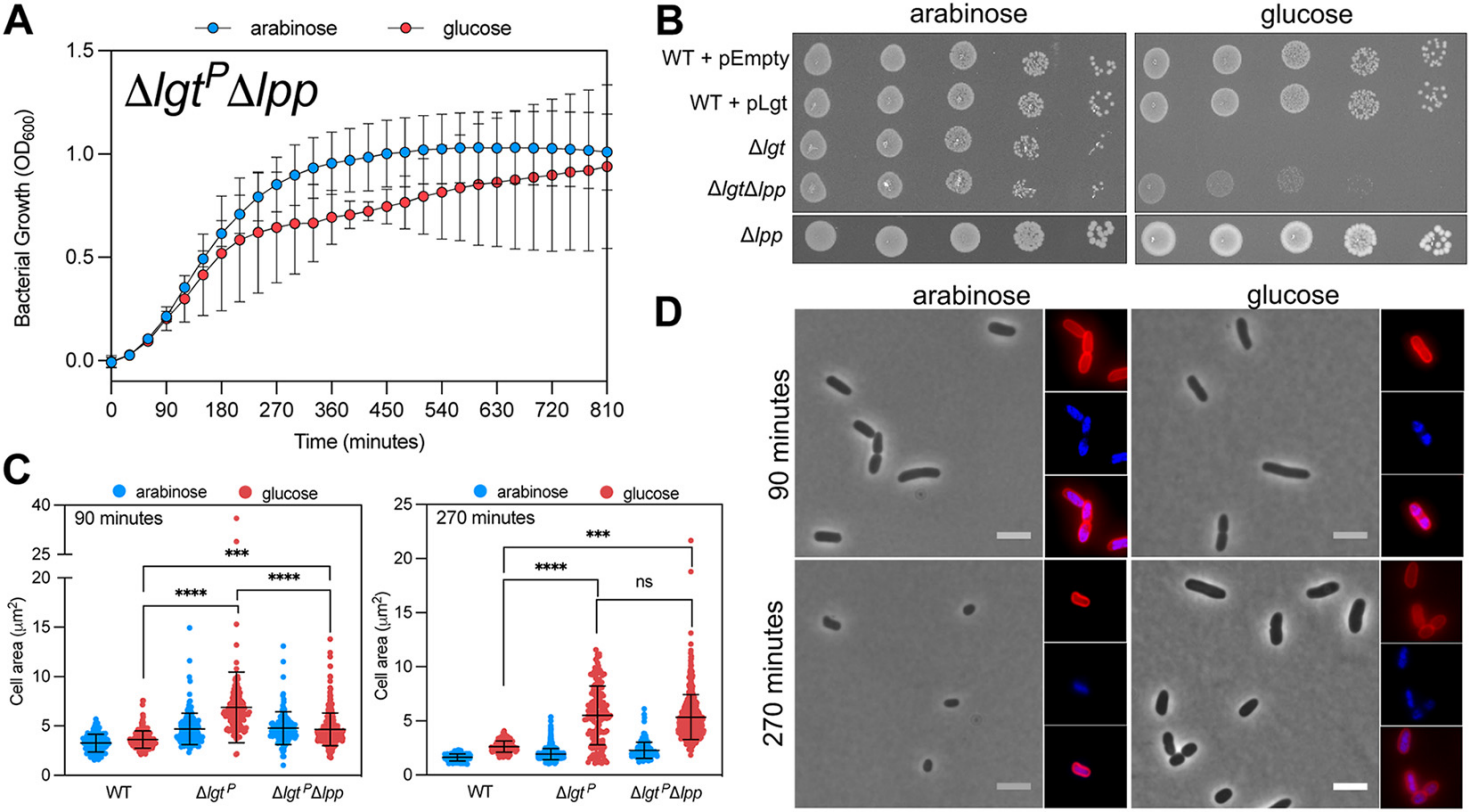
Lpp deletion partially restores growth of an Lgt depletion strain. (A) Strain PAP9403 *lpp*::Tn*10* (Δ*lgt*^P^ Δ*lpp*) was grown in LB medium containing 0.2% l-arabinose or 0.2% d-glucose. Graphed data represent duplicate OD_600_ measurements of biological triplicates. (B) Spot dilution assay of cultures of BW25113 + pEmpty, BW25113 + pLgt, Δ*lgt*^P^, Δ*lgt*^P^ Δ*lpp*, and Δ*lpp* were washed with LB lacking sugar after overnight growth and grown for 2 h in LB. Cells were then spotted on plates as 5 μL of 10^−1^ to 10^−5^ dilutions. (C) Cell area measurements (*n* > 100 cells) from corresponding strains were performed on phase-contrast images using Microbe J. The WT is BW25113. ****, *P* < 0.0001; ***, *P* < 0.001. (D) Phase-contrast images of Δ*lgt*^P^ Δ*lpp* grown in the presence of 0.2% l-arabinose or 0.2% d-glucose. Nucleoids were stained with Hoechst (blue) and the membrane was stained with FM4-64X (red). Bar, 5 μm.

Lpp is the most abundant lipoprotein in E. coli and therefore a prominent substrate for the lipoprotein modification enzymes. Since very low levels of Lgt are sufficient for growth, we hypothesized that deletion of *lpp* would fully restore growth and morphology upon Lgt depletion. The double mutant Δ*lgt*^P^Δ*lpp* grew in l-arabinose and d-glucose, but growth was highly variable and the optical density at 600 nm (OD_600_) was lower than that for Δ*lgt*^P^ at 270 min (Fig. 3). In a spot dilution assay, colonies were smaller in Δ*lgt*^P^ Δ*lpp* compared to full expression of *lgt*, but CFU were constant, independent of l-arabinose concentration (see Fig. S3). The morphology of Δ*lgt*^P^Δ*lpp* was less severe compared to Δ*lgt*^P^ in d-glucose, but cells were larger than wild-type cells upon entry into stationary phase (270 min) (Fig. 2). In stationary phase, the percentage of Lpp cross-links to peptidoglycan increases (28), which might explain the observed morphology. Growth of the depletion strain was thus partially restored in the absence of Lpp and viability increased. To test whether Lgt is essential in the absence of Lpp, we first tried to cure pLgt from Δ*lgt*^P^ Δ*lpp* by using the pFREE system. Whereas pLgt was readily removed from a wild-type background, the plasmid could not be lost from Δ*lgt*^P^Δ*lpp* (see Fig. S2). Attempts to delete *lgt* and replace it with a kanamycin cassette by P1 transduction in a *lpp*::Tn*10* strain were also unsuccessful. Together, these results suggest that basal levels of *lgt* expression from pLgt, alongside the absence of *lpp*, is sufficient to sustain growth.

### Deletion of Lpp does not restore viability of the chromosomally encoded *lgt* depletion strain.

**FIG 4:**
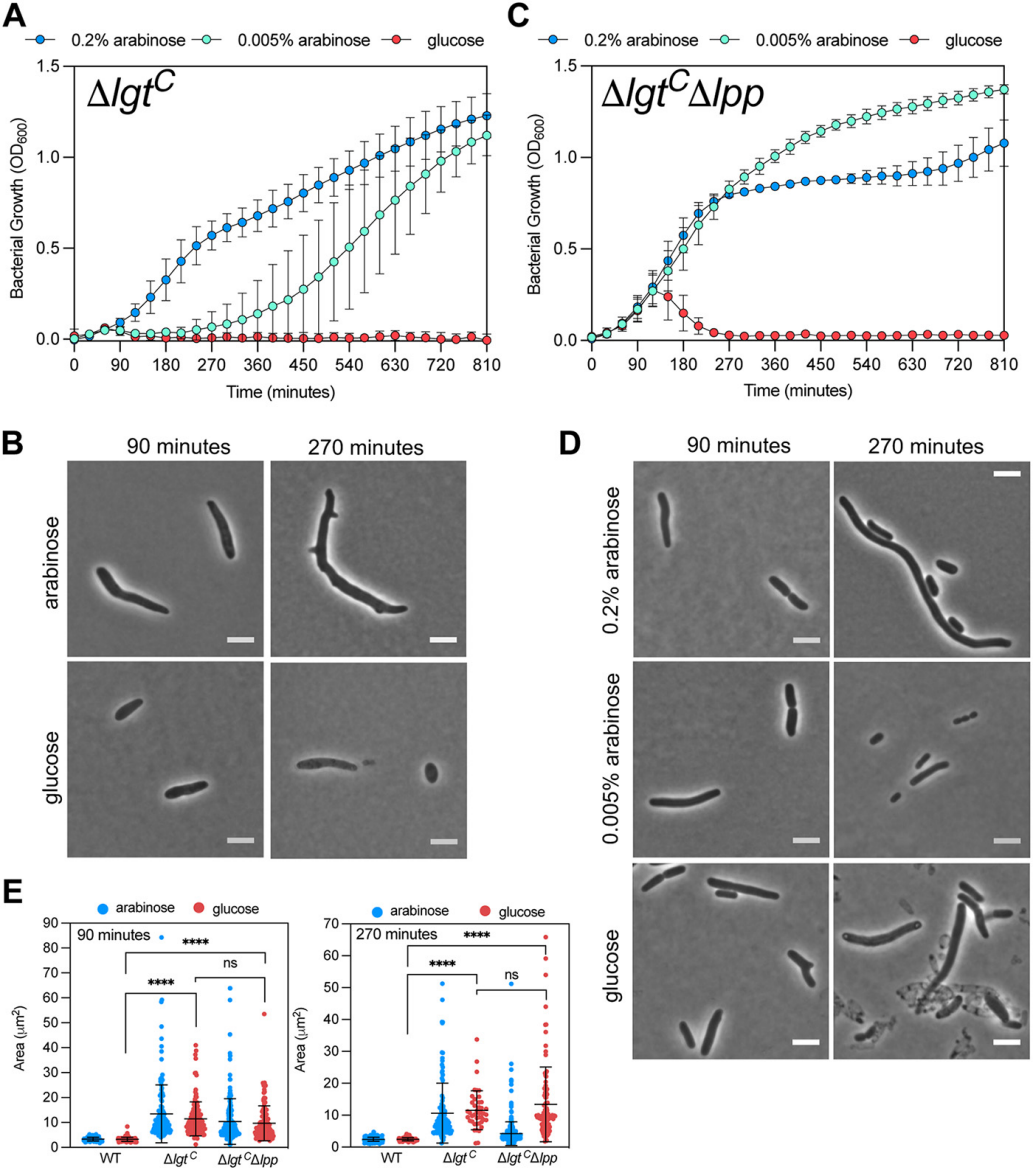
Deleting *lpp* does not rescue the distorted morphology caused by low Lgt levels. (A) Growth of chromosomal *lgt* depletion strain (MG1655Δ*lgt*) (Δ*lgt*^C^) in 0.2% and 0.005% l-arabinose or 0.2% d-glucose. (B) Phase-contrast microscopy images of Δ*lgt*^C^. (C) Growth of Δ*lgt*^C^ Δ*lpp* in high (0.2%) or low (0.005%) l-arabinose or in 0.2% d-glucose. (D) Phase-contrast microscopy images of Δ*lgt*^C^ Δ*lpp*. Bar, 5 μm. (E) Cell area measurements of cells (*n* > 100) from corresponding strains were performed on phase-contrast images using Microbe J. WT was MG1655.

As the basal level of expression of *lgt* from the multicopy pLgt may be a factor in the survival of the depletion strain, we sought to reduce the copy number of *lgt*. Diao et al. (29) recently showed that low levels of Lgt are sufficient to maintain cell viability. They described the construction of a MG1655 strain in which *lgt* was under the control of an arabinose promoter on the chromosome at the lambda attachment site (λatt; Δ*lgt*^C^). When grown under the same conditions as Δ*lgt*^P^ in liquid media, Δ*lgt*^C^ was unable to grow in the presence of glucose and showed greater sensitivity to reduced arabinose concentrations (Fig. 4; see also Fig. S2). Interestingly, very low concentrations of l-arabinose (0.005%) were sufficient for growth, albeit after a dose-dependent lag period (see Fig. S2). Cells grown in the presence of 0.2% l-arabinose displayed distorted cell morphology with filamentous cells possessing multiple cell poles (Fig. 4). In the absence of Lpp, the depletion strain grew in d-glucose for 135 min before growth declined and was not further restored (Fig. 4). The cells grew as filaments that lysed after 270 min. These results showed that Lpp is not the only cause of cell death in the absence of Lgt.

### Complementation of Δ*lgt*^C^ is observed upon *lgt* expression in *trans* from a low-copy-number plasmid.

**FIG 5:**
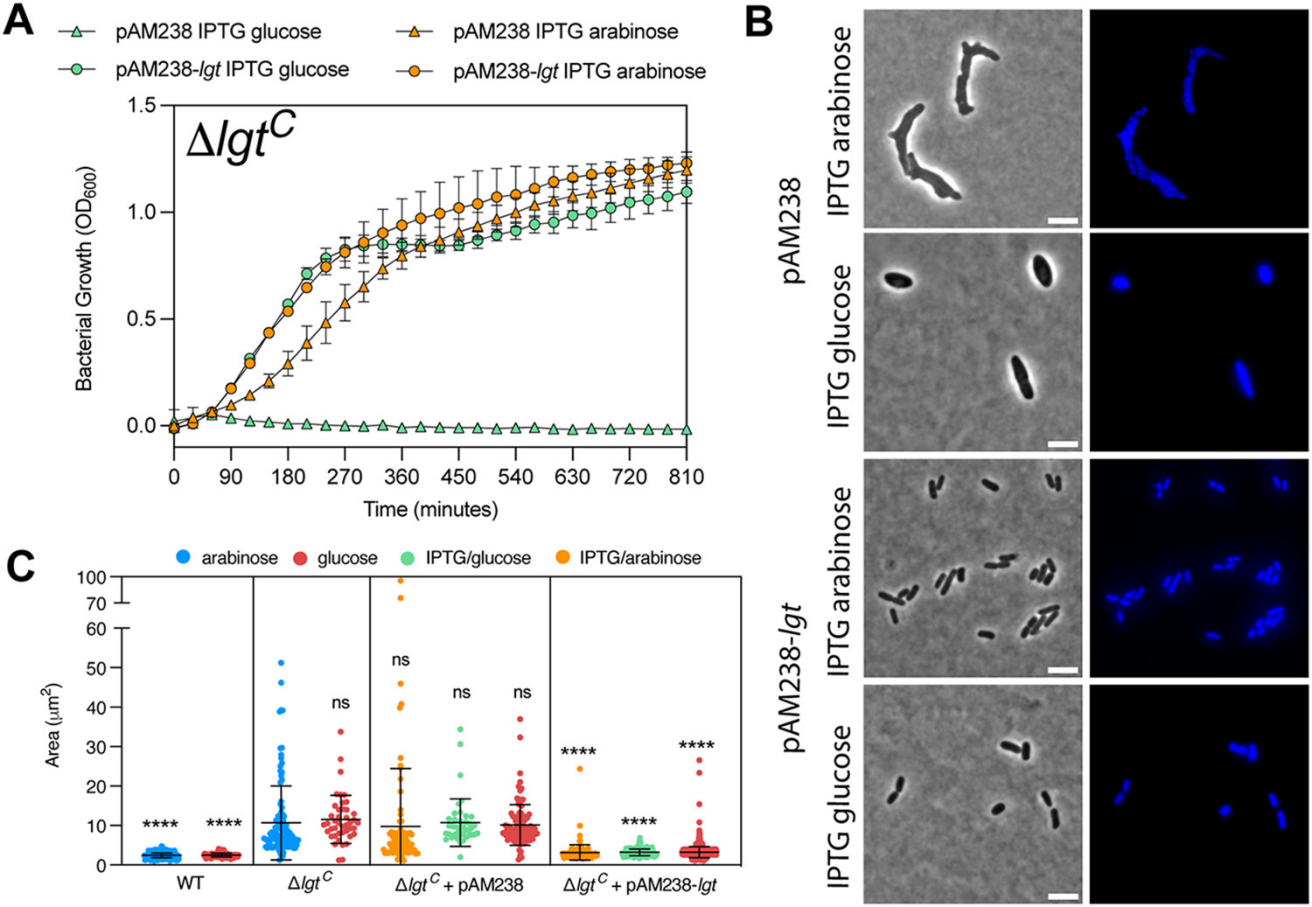
Complementation of MG1655Δ*lgt* by Lgt from pAM238-*lgt*-*flag*_3_. (A) Growth of chromosomal *lgt* depletion strain (Δ*lgt*^C^) containing empty plasmid pAM238 (triangle; SLEC70) or pAM238-*lgt*-*flag*_3_ (round; SLEC71) in the presence of 0.2% l-arabinose with 5 mM IPTG (orange) and 0.2% d-glucose with 5 mM IPTG (green). (B) Phase-contrast images of SLEC71 and SLEC70 from 270 min. DNA was stained with Hoechst. Bar, 5 μm. (C) Cell area measurements of cells (*n* = 100) from corresponding strains at 270 min were performed on phase-contrast images using Microbe J. Statistical comparisons were in relation to Δ*lgt*^C^ in the presence of 0.2% l-arabinose. ****, *P* < 0.0001; ns, no significance.

Our results suggested that Lgt levels were too low to restore a wild-type morphology in Δ*lgt*^C^ grown in l-arabinose. Indeed, expression of *lgt* from an isopropyl-β-d-thiogalactopyranoside (IPTG)-inducible promoter on a low-copy-number plasmid (pAM238-*lgt*-*flag*_3_) restored cell size and morphology both in the presence of l-arabinose and d-glucose with clearly distinguishable nucleoids (Fig. 5). This indicated that only Lgt is responsible for the aberrant phenotypes observed under depletion conditions.

## DISCUSSION

The correlation between lipoprotein modification and the cellular localization and function of the major lipoprotein Lpp in E. coli has been known for many years. Henry C. Wu and colleagues identified the *lgt* gene in Salmonella enterica serovar Typhimurium through analysis of a library of temperature-sensitive mutants and showed that deletion of *lpp* rescued the temperature-sensitive phenotype of an *lgt*(ts) mutant (5). The observation that Lgt is highly conserved in bacteria while Lpp is conserved in many but not all proteobacteria (30) raised the question of whether Lpp is important for the essentiality of Lgt in bacterial viability. Furthermore, since the lipoprotein modification pathway is unique to bacteria and the enzymes have domains exposed to the periplasm or exterior of the cell that seem readily accessible to small molecules, this pathway is potentially a good target for the development of novel antibiotics.

**FIG 6:**
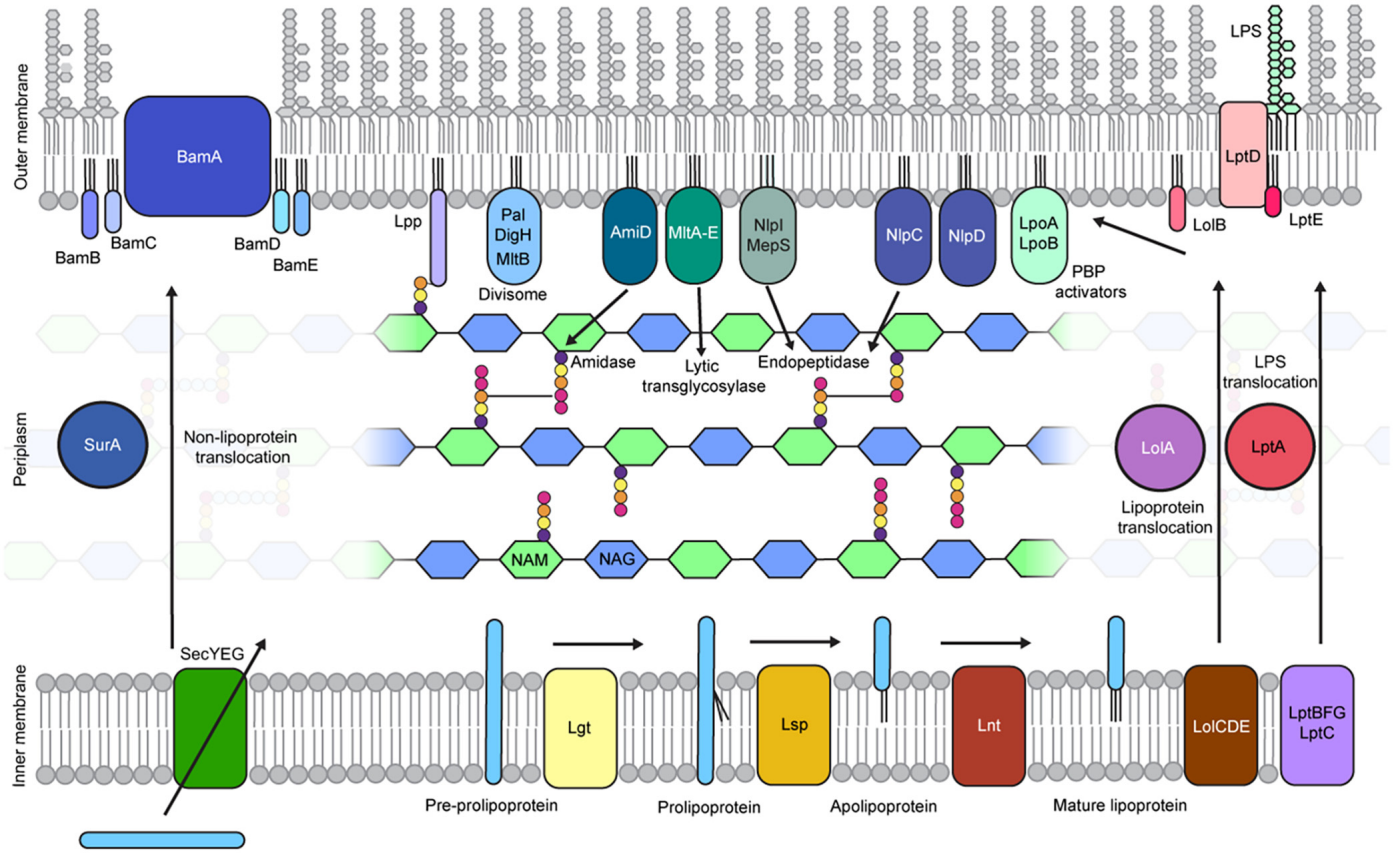
Essential cell envelope biogenesis processes that depend on correct outer membrane localization of lipoproteins. About 20 lipoproteins are involved in essential envelope biogenesis processes in E. coli, including peptidoglycan biogenesis and cell division. Three lipoproteins are essential, i.e., insertion of lipoproteins in the outer membrane is dependent on the Lol machinery (LolB), insertion of outer membrane β-barrel proteins (OMPs) is dependent on the Bam machinery (BamD), and assembly of LPS depends on the Lpt system (LptE). The implications for lipoproteins in cell wall biogenesis and cell division are discussed in the main text.

Recent phenotypic screenings have identified inhibitors targeting signal peptidase II (Lsp) (23) and downstream processes that involve transport of lipoproteins to the outer membrane (Lol) (21, 22). In all studies, absence of Lpp led to a decrease in efficacy of inhibition. The recent paper by Diao et al. demonstrated that inhibition of Lgt by a synthetic cyclic peptide leads to morphology changes, membrane blebbing, and reduced viability (29). The effect of Lgt inhibition is, however, not reversed by *lpp* deletion. Cell size increase is modest in the presence of inhibitor compared to the Lgt depletion strain grown under restrictive conditions. These findings raise the question of whether the cyclic peptide directly targets Lgt or other off-target cell envelope components; however, inhibiting enzyme activity is different from lacking the enzyme, which probably leads to a more drastic phenotype. We took advantage of two Lgt depletion strains to study the role of Lpp on cell morphology and viability when cells were depleted for Lgt. In both strains, *lgt* expression is under the control of the arabinose-inducible P_ara_ promoter but differs in copy number of the *lgt* gene, i.e., one strain depends on a multicopy plasmid to control *lgt* expression, while the other strain contains a single chromosomal *lgt* gene. Deletion of endogenous *lgt* was performed similarly in both strains and did not affect downstream expression of *thy*A (25, 29). Depletion of Lgt led to a distorted morphology, leakage of DNA, cell lysis, and loss of viability. Wu and colleagues observed swollen, oval, and lysed cells in the *lgt*(ts) strain of Salmonella when grown at nonpermissive temperature (5). Strikingly, the morphology of the chromosomally controlled Lgt depletion strain was affected when grown under permissive conditions, in the presence of l-arabinose, and cells displayed branched cell poles. This phenotype was also observed, although more severely, in a mutant of E. coli lacking *dac*A, which encodes d,d-carboxypeptidase penicillin binding protein 5 (PBP5) (31). A similar *lgt* conditional null mutant in a uropathogenic E. coli strain led to retraction of the cytoplasmic membrane, membrane blebbing, and permeabilization of the outer membrane upon Lgt depletion, which was different from results with the K-12 wild-type strain used in that study, where DNA leakage and cell lysis were clearly observed (29). The genome has multiple pathogenicity islands inserted into its genome that may affect nucleoid organization and gene expression in general (32). Prolonged growth under restrictive conditions led to a revertant phenotype in the plasmid-controlled Lgt depletion strain. The survival was likely due to arabinose-independent low expression of *lgt*, since chromosomal mutations were not observed and the plasmid could not be lost from the strain, although the presence of an additional factor leading to growth restoration could not be excluded. Diao et al. showed a greater loss in viability of Δ*lgt*^C^ after 5 h of growth in the absence of Lpp when low concentrations of arabinose were present. After a short period of depletion, we observed no change in viability between Δ*lgt*^C^ and Δ*lgt*^C^ Δ*lpp* (see Fig. S3 in the supplemental material), but we did observe improved growth in liquid medium for the depletion strain when Lpp was removed and Lgt was expressed in low concentrations of arabinose (Fig. 4). In each instance, we showed that deletion of Lpp did not rescue growth of the chromosomally controlled Lgt depletion strain when Lgt was not expressed and that Lgt was therefore essential in E. coli independently of Lpp. Altogether, our findings show that other lipoproteins are important for cell viability in E. coli. Detailed roles have been assigned to the essential lipoproteins LolB, BamD, and LptE. LolB serves as a lipoprotein receptor in the outer membrane and is part of the Lol machinery (11). BamD is one of the four lipoproteins that constitute the Bam machinery involved in assembly of β-barrel proteins in the outer membrane (14, 15). LptE is important for correct assembly of LPS on the cell surface (12, 13). Lpp is the most abundant protein in the cell envelope of E. coli. In the absence of Lpp, substrate competition for these essential lipoproteins by Lgt is thus reduced, leading to fitness upon Lgt depletion, as previously suggested by Wu and colleagues (33). Over recent years, functional insight has been obtained about other lipoproteins involved in cell envelope biogenesis, including cell division, outer membrane biosynthesis, cell wall (peptidoglycan) biosynthesis, and envelope integrity; however, these lipoproteins do not have clearly distinguished roles in each of these processes (Fig. 6). For example, Lpp, the first outer membrane lipoprotein identified (34) that covalently links the outer membrane to the cell wall (35), regulates mechanical properties of the E. coli cell envelope (36), determines the space between the outer membrane and peptidoglycan (37), and has a role in positioning large envelope-spanning complexes (38). A recent report described the role of Lgt in the correct function of DcrB, a lipoprotein in the cytoplasmic membrane which, together with small integral membrane protein YciB, functions in maintenance of membrane fluidity (39). Cell division requires the coordination of cell wall synthesis and membrane invagination. Two outer membrane lipoproteins, LpoA and LpoB, stimulate enzymatic activity of PBP1A and PBP1B, respectively, and their combined loss is lethal (40–42). In fact, when LpoB is localized to the cytoplasmic membrane, rod shape is lost (43). Amidases cleave the bond between the *N*-acetylmuramic acid sugar moiety and l-alanine of the peptide stem in peptidoglycan. AmiC is activated through interaction with lipoprotein NlpD and splits the peptidoglycan between daughter cells (44). Lipoprotein AmiD is an amidase with broad specificity, cleaving both muropeptides and intact peptidoglycan (45). NlpI is a general adaptor protein for peptidoglycan hydrolases (46) and interacts with, among other lipoproteins, MepS, an outer membrane lipoprotein with d,d-endopeptidase activity (47). Although not essential by itself, the lipoprotein Pal plays an important role as part of the Tol-Pal complex in maintaining cell envelope integrity, targeting of cell division components to mid-cell, and outer membrane constriction (48–51). Several lytic transglycosylases (MltA to -E) are lipoproteins involved in peptidoglycan biogenesis (52–56). MltB and DigH, a novel glycosyl hydrolase (57), are directly linked to the lipoprotein Pal, which noncovalently interacts with peptidoglycan (58) upon its dissociation from TolB (49). Thus, lipoproteins have a central function in cell wall biogenesis. Slowly, evidence has been obtained on how the different complexes operate and are coordinated. Finally, two major envelope stress response systems in E. coli depend on outer membrane lipoproteins: the Rsc system on RcsF (59) and the Cpx system on NlpE (60). Mislocalization of RscF and NlpE leads to activation of downstream two-component systems and expression of repair genes (61). In conclusion, the increase in knowledge on lipoproteins with a function in cell envelope biogenesis shows that this very diverse class of fatty acid acylated proteins are important for bacterial physiology. Thus, removal or reduction of Lgt and therefore reduced lipoprotein processing have a multifaceted effect on the cell envelope. As the first enzyme of the posttranslational modification pathway, Lgt is essentially independent of the major lipoprotein Lpp and therefore a promising target for the development of novel antibiotics.

## MATERIALS AND METHODS

### Bacterial growth conditions.

Bacterial cells were grown in Luria-Bertani broth (LB) or agar (LBA) at 37°C. When required, kanamycin (Kan, at 40 mg/liter), chloramphenicol (Cm, at 30 mg/liter), spectinomycin (Spec, at 50 mg/liter), or tetracycline (Tet, at 10 mg/liter) was added. A complete list of strains used in this study can be found in Table S1 in the supplemental material. Both Δ*lgt*^P^ and Δ*lgt*^C^ strains were grown in the presence of 0.2% l-arabinose unless stated otherwise. Overnight and initial growth were conducted as follows: single colonies of the required strains were selected and grown overnight in LB. Overnight cultures were washed three times in fresh LB and diluted 1/100 in LB without l-arabinose or IPTG to deplete Lgt.

### Generation of *lpp* mutants.

*lpp* deletion mutants were created by P1 transduction of *lpp*::Tn*10* from PAP8505 (19). The donor strain was grown in LB with 0.2% l-arabinose until an OD_600_ of 0.1 was reached. Different dilutions of P1 phage stock were added, and strains were grown until cell lysis was observed. Cleared P1 lysate from the lowest concentration of phage was used for transduction. Recipient strains were grown in liquid medium containing 0.2% l-arabinose overnight. Cells (100 μL) were supplemented with 10 mM CaCl_2_ and dilutions of P1 phage were added and incubated without shaking at 37°C for 30 min. Sodium citrate was added to a final concentration of 150 mM to chelate Ca^2+^. LB medium (1 mL) was added, and cells were grown at 37°C for 1 h while shaking. Cells were plated on Tet LBA containing Na-citrate to select for *lpp*::Tn*10* transductants. Colonies were restreaked once on Na-citrate Tet plates to rid P1 phage.

### Construction of plasmid pAM238-*lgt-flag_3_*.

The upperFLAG and lowerFLAG primers (Proligo) were annealed and inserted into plasmid pCHAP9246 digested with restriction enzymes XbaI and HindIII to create pAM238-*lgt-flag_3_*.

### Growth kinetics.

For growth kinetics, overnight and initial growth were conducted as described above for bacterial growth conditions. Cultures were diluted to an approximate final OD_600_ of 0.05 in 96-well plates with l-arabinose (0.2% and 0.005%), d-glucose (0.2%), and/or IPTG (5 mM). The plates were incubated at 37°C, and the OD_600_ was measured in a Tecan plate reader. Each condition was conducted with three biological replicates with at least two technical replicates.

### Spot dilution assay.

For spot dilution assays, overnight and initial growth were conducted as described above (Bacterial growth conditions). Cultures were diluted to a final OD_600_ of approximately 0.1. The adjusted cultures were serially diluted 10-fold in LB, and 5 μL was spotted onto LB agar plates containing decreasing concentrations of l-arabinose (0.2%, 0.05%, 0.0125%, 0.0031%, 0.008%, 0.004%, 0.002%), d-glucose (0.2%), or nonsupplemented LBA. Where required, LBA-Cm was used. Plates were incubated overnight at 37°C and imaged, and CFU counts per milliliter were recorded.

### Microscopy.

Overnight and initial growth were conducted in batch cultures. Cultures were diluted to a final OD_600_ of approximately 0.05. Cultures were grown for a further 270 min at 37°C with 0.2% l-arabinose, 0.005% l-arabinose, 0.2% d-glucose, or 5 mM IPTG (or a combination of conditions). Samples were taken at 90 min and 270 min, and the cells were fixed in 2.8% paraformaldehyde, 0.04% glutaraldehyde and incubated at room temperature (RT) for 15 min. Fixed cells were washed 3 times by centrifugation at 13,200 × *g* and resuspended in phosphate-buffered saline (PBS). For fluorescent labeling, 10 mg/liter Hoechst 33342 and 0.2 mg/liter FM 4-64FX (Invitrogen) were added, and mixtures were incubated for 15 min at RT in the dark. Cells were washed 3 times in PBS. Microscope slides were prepared with agarose pads (1% agarose in H_2_O), and 5 μL of cells was added. Imaging was performed on a Zeiss Axio Observer microscope, and images were analyzed with the ImageJ plugin MicrobeJ. Final images were adapted for publication with ImageJ and Adobe Illustrator.

### Collection of revertants.

Cultures from growth kinetics of Δ*lgt*^P^ grown in 0.2% d-glucose were restreaked on LBA-Cm and incubated overnight at 37°C. A single colony from each of the nine samples collected was selected and stored (Δ*lgt*^P^ m1 to Δ*lgt*^P^ m9).

### Whole-genome sequencing.

DNA samples were fragmented with a Covaris M220 focused ultrasonicator (Covaris Ltd., Brighton, United Kingdom) using microTUBE-15 to 350 bp. The TruSeq DNA PCR-free libraries prep kit (Illumina) was used, following the instructions of the kit manufacturer. Sequencing was carried out on an Illumina NextSeq 500 platform. Raw reads were processed with an in-house bioinformatics pipeline for quality (62). The genome of the strain Escherichia coli Δ*lgt*^P^ was assembled using SPAdes (63). An automatic annotation was completed with Prokka (64) for the strain E. coli BW25113 (accession number CP009273.1) as a reference. Then, we used the variant calling pipeline (v0.11.0), which is available online (https://github.com/sequana/variant_calling). Analysis of variants was further performed using Geneious Prime.

### Glycine-SDS and Tricine-SDS-PAGE and immunoblotting.

For the detection of Lgt-c-myc_2_ from pLgt and pLgt^m1^ to pLgt^m3^, plasmids were extracted from Δ*lgt*^Pm1^ to Δ*lgt*^Pm3^ using a Qiagen Miniprep kit, transformed into chemically competent BW25113 cells, and grown overnight on LBA-Cm. Single colonies were selected and grown overnight in LB-Cm. Overnight cultures were diluted 1/100 in LB-Cm and supplemented with l-arabinose (0.2%, 0.005%), or d-glucose (0.2%). Cultures were grown for a further 3 h at 37°C. Whole-cell pellets were taken and resuspended in sample buffer (10% glycerol, 2.5% SDS, 100 mM Tris [pH 8], 10 mM dithiothreitol, phenol red) equivalent to 0.01 OD_600_ units/μL before heating at 100°C for 10 min. Samples were loaded onto 4 to 15% Mini-Protean TGS stain-free precast protein gels (Bio-Rad), and proteins were separated by migration at a constant amperage (20 mA). Proteins were transferred to nitrocellulose membranes by using a Bio-Rad TurboBlot. Membranes were briefly stained in Ponceau S before washing in H_2_O. Membranes were then blocked overnight in PBS–5% milk. Primary THE c-Myc tag monoclonal antibodies (Genscript) were diluted 1:10,000 in PBS–1% bovine serum albumin–0.05% Tween 20 and incubated for 1 h at RT. The blots were washed in PBS–0.1% Tween 20 twice for 5 min and three times for 10 min. Secondary anti-mouse-horseradish peroxidase (HRP) antibodies were diluted 1:10,000 in PBS–5% milk, and blots were incubated for 1 h. The blots were washed as described above. SuperSignal West Femto maximum sensitivity substrate was used to detect c-*myc*_2_-tagged proteins when imaged under chemiluminescence and colorimetric conditions on a ChemiDoc imager (Bio-Rad).

For the detection of Lpp, cultures were diluted to a final OD_600_ of approximately 0.1. Cultures were grown for a further 270 min at 37°C in LB with 0.2% l-arabinose, 0.2% d-glucose, or nonsupplemented. Proteins from 1-mL cell cultures were precipitated in 10% ice-cold trichloroacetic acid and incubated on ice for 30 min. Precipitated samples were centrifuged at 13,200 × *g* for 5 min and washed twice in ice-cold (−20°C) acetone. After centrifugation, the pellet was air dried and resuspended in Tricine sample buffer (Bio-Rad) to an equivalent of 0.01 OD_600_ units/μL before heating at 100°C for 10 min. Samples were loaded onto Invitrogen Novex 16% Tricine protein gels and migrated at constant voltage (100 V) for 4 h. Western blots were blocked in Tris-buffered saline (TBS)–5% milk–0.1% Tween 20, primary anti-Lpp antibodies were diluted 1:20,000 in TBS–1% milk, and secondary anti-rabbit-HRP was diluted 1:10,000 in TBS–0.1% Tween 20. Detection was conducted as describe above.

### pFREE plasmid curing.

pFREE plasmid curing was performed as previously described (27). Briefly, overnight cultures of BW25113 pLgt, Δ*lgt*^P^ Δ*lpp*, or Δ*lgt* revertants were diluted 1/100 in LB and grown for 1 h at 37°C. Cultures were then made chemically competent and 50 ng of pFREE was transformed. After 2-h incubation in LB at 30°C, the cultures were induced by diluting 1/20 in LB containing 0.2% rhamnose, 200 ng/μL anhydrotetracycline, 50 mg/liter Kan, and further incubated overnight at 37°C. Uninduced strains were used as negative controls. The next day, cultures were plated onto LB and LB-Cm and the curing efficiency was calculated by determining the ratios in colony numbers. When colonies were susceptible to Cm, presence or absence of pLgt was confirmed by PCR with pBAD_F and pBAD_R primers (see Table S1).

### Statistical analysis and data presentation.

All numerical data were analyzed with GraphPad Prism (version 9.3.0). Microscopy images presented here were prepared with ImageJ and Adobe Illustrator. For comparisons of cell areas, distribution was determined by a Shapiro-Wilk test, and one-way analysis of variance with multiple comparisons by the Kruskal-Wallis test was employed.

### Data availability.

WGS data have been submitted to the NCBI SRA database under accession number PRJNA860548.
