# Supplementary material for: A Defect in Lipoprotein Modification by Lgt Leads to Abnormal Morphology and Cell Death in Escherichia coli That Is Independent of Major Lipoprotein Lpp
Source: J Bacteriol. 2022 Aug 8;204(9):e00164-22. doi: 10.1128/jb.00164-22 (PMC9487459; doi:10.1128/jb.00164-22)
Supplement: Supplemental file 1 — Fig. S1 to S3 and Table S1. Download jb.00164-22-s0001.pdf, PDF file, 1.4 MB [file jb.00164-22-s0001.pdf]

Supplementary Figures and Table

Fig. S1

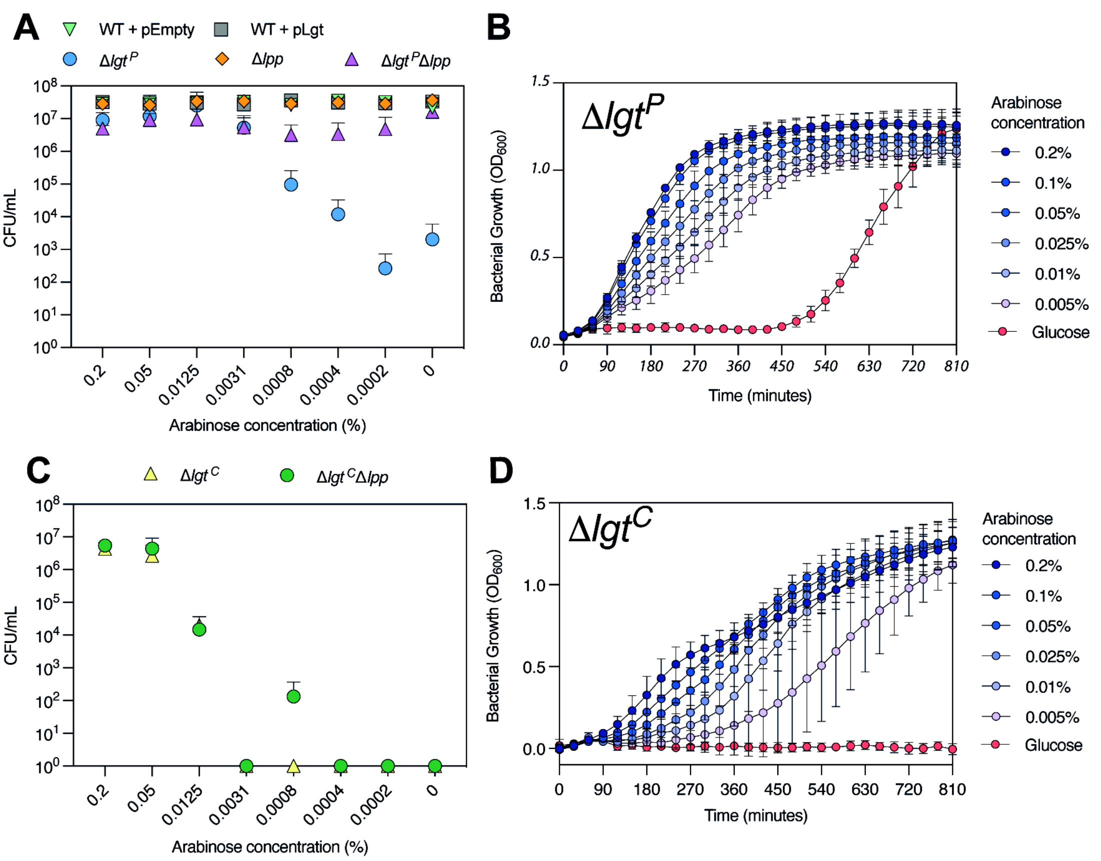

Fig. S1. Whole genome sequencing of Lgt revertant plasmids.

(A) Sequence of P_{ara} from $pLgt^{m1\text{-}2}$ containing a single nucleotide polymorphism (SNP) G-A at residue 210. (B and C) Nucleotide and translated sequence of *myc*$_2$-tag region of $pLgt^{m1\text{-}9}$. Red box indicates stop codons, green box indicates deletions relative to pLgt.

Fig. S2.

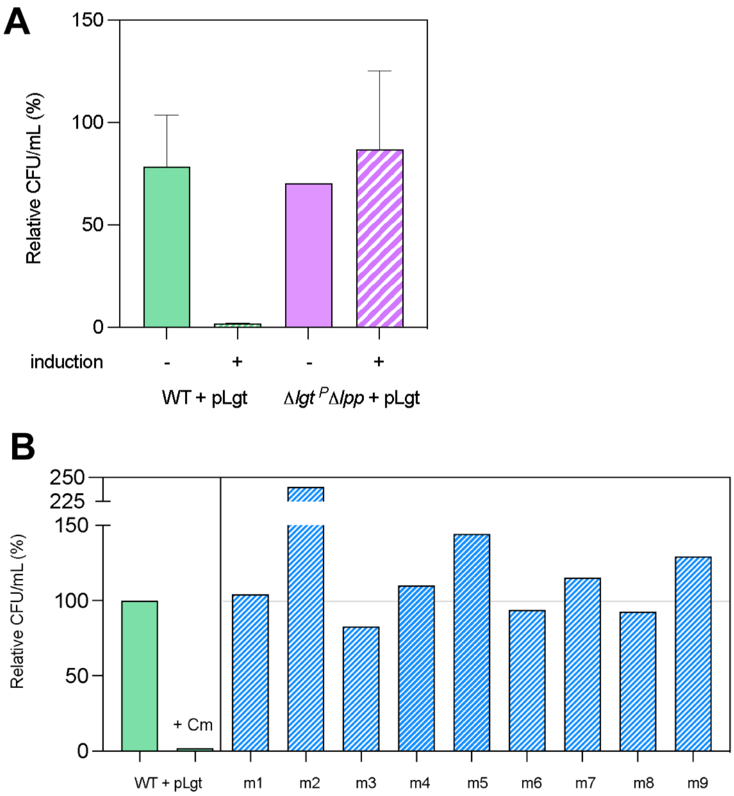

Fig. S2. Lgt complementing plasmid cannot be counter selected from *lgt* depletion strain lacking Lpp.

Cas9/gRNA encoding plasmid pFREE targeting the origin of replication of plasmids was transformed into wild type strain BW25113 containing (A) pCHAP9224 (pLgt) and Δ*lgt*^{P}Δ*lpp* or (B) Δ*lgt*^{P} revertants m1-9. Expression of *cas9/gRNA* was induced with 0.2% L-arabinose and clones are selected on non-selective plates and chloramphenicol (25 mg/L) containing plates. Colonies were counted in the two strains without and with induction of the Cas9/gRNA system. Loss of pLgt was expressed as ratio of number of colonies on non-selective and Cm plates. Where present, error bars indicate standard deviation from the mean at least two replicates, otherwise n = 1.

Fig. S3.

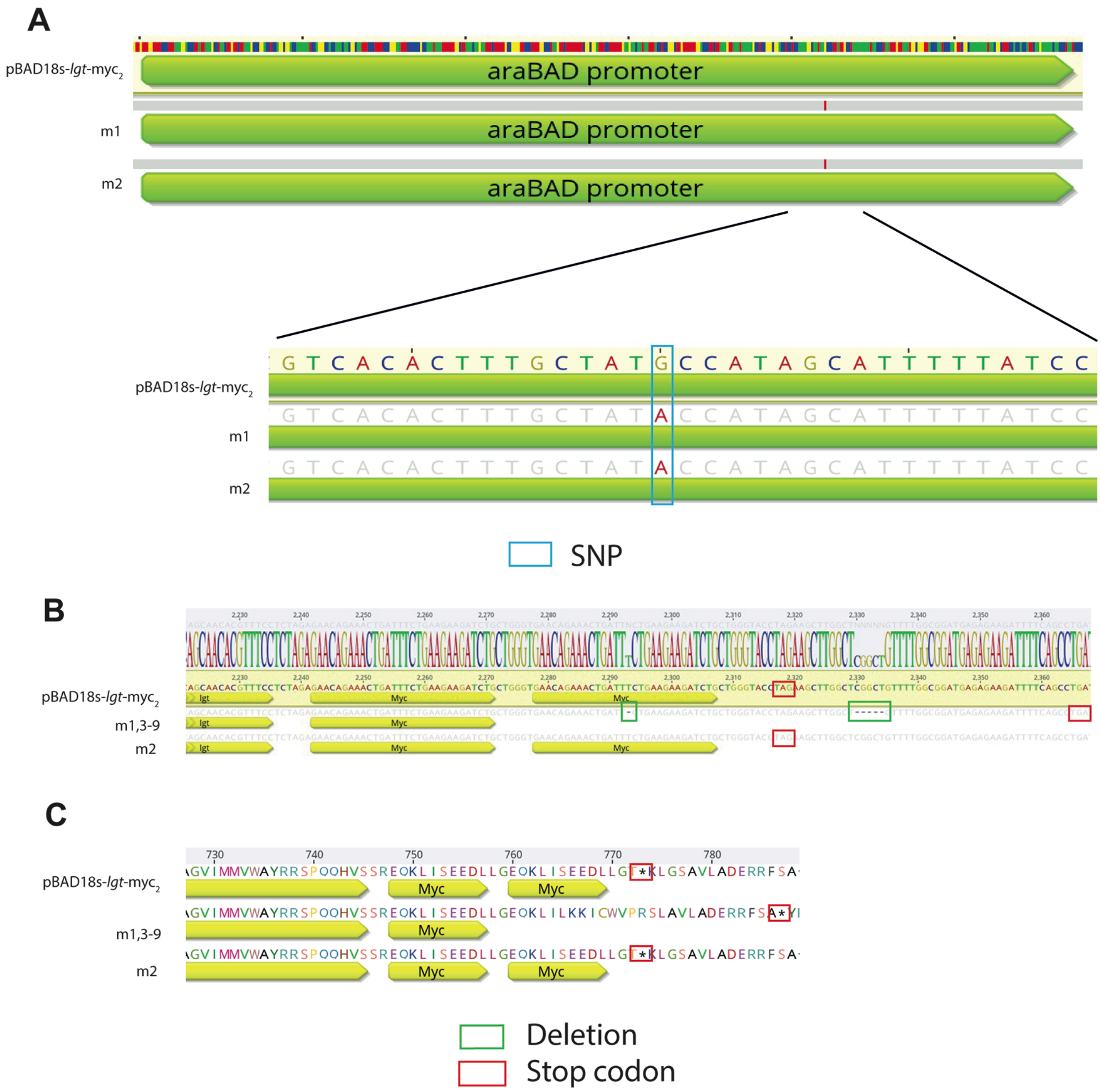

Fig. S3. Growth and viability of Lgt depletion strains.

(A) Colony forming units (CFU) of Δ*lgt*^{P} compared to BW25113 and Δ*lpp* derivative, and overexpression strain BW25113 containing pCHAP9224 versus empty pBAD18s-Cm; or (C) of chromosomal *lgt* depletion strain (Δ*lgt*^{C}) compared with Δ*lpp* derivative. (B) Growth kinetics of depletion strain Δ*lgt*^{P} ; or (D) of Δ*lgt*^{C} in varying L-arabinose concentrations and 0.2% D-glucose. Time t=0 corresponds to 2 hours of growth in LB medium without sugar. Graph represents duplicate OD_{600} measurements of biological triplicates (n=3).

Table S1. Strains, plasmids and primers used in this study.

Name	Alt Name	Description	Reference
**Strains**			
BW25113	SLEC30	*E. coli* K-12 $lacI^{q}$ $rrnB_{\mathrm{T14}}$ $\Delta lacZ_{\mathrm{WJ16}}$ *hsdR514* $\Delta araBAD_{\mathrm{AH33}}$ $\Delta rhaBAD_{\mathrm{LD78}}$	(1)
BW25113 Δlgt^{P}	PAP9403	BW25113 Δ*lgt*::$\mathrm{Kan^r}$ + pBAD18s-Cm-lgt^{WT}-c-myc_2	(2)
BW25113 $\Delta lgt^{\mathrm{P}}\Delta lpp$	SLEC44	BW25113 Δ*lgt*::$\mathrm{Kan^r}$ *lpp*::Tn10 + pBAD18s-Cm-lgt^{WT}-c-myc_2	This study
BW25113 pEmpty	SLEC45	BW25113 + pBAD18s-Cm	This study
BW25113 pLgt	SLEC22	BW25113 + pBAD18s-Cm-lgt^{WT}-c-myc_2	This study
PAP8505		BW25113 *ybeX*-(*kan-rpoCter*-p_{araB})-*lnt* *lpp*::Tn10	(3)
BW25113 Δ*lpp*	SLEC65	BW25113 *lpp*::Tn10	This study
BW25113 Δlgt^{P} m1-9		BW25113 Δ*lgt* revertants	This study
MG1655	SLEC69	F-, λ-, *ilv*G- *rfb-50 rph-1*	Lab collection
MG1655 Δlgt^{C}	SLEC67	MG1655 Δ*lgt*::$\mathrm{Kan^r}$ λattB-pBAD-*lgt*	(4)
MG1655 $\Delta lgt^{\mathrm{C}}\Delta lpp$	SLEC68	MG1655 Δ*lgt*::$\mathrm{Kan^r}$ λattB-pBAD-*lgt*Δ*lpp*::Tn10	This study
MG1655 Δlgt^{C} pEmpty	SLEC70	MG1655 Δ*lgt*::$\mathrm{Kan^r}$ λattB-pBAD-*lgt* + pAM238	This study
MG1655 Δlgt^{C} pLgt	SLEC71	MG1655 Δ*lgt*::$\mathrm{Kan^r}$ λattB-pBAD-*lgt* +	This study

		pAM238-*lgt-flag*$_3$	
**Plasmids**			
pBAD18s-Cm		pBR322 origin, P*ara* promoter (pBAD), Cm^r	(2)
pBAD18s-Cm-*lgt*^{WT}-c-*myc*$_2$	pCHAP9224, pLgt	pBR322 origin, P*ara* promoter (pBAD), Cm^r, expressing *E. coli lgt*	(2)
pLgt^{m1-9}		pBAD18s-Cm-*lgt*^{WT}-c-*myc*$_2$	This study
pAM238		pSC101 origin, Plac promoter, Spc^r	(5)
pAM238-*lgt-myc*$_2$	pCHAP9246	pSC101 origin, Plac promoter, Spc^r, expressing *E. coli lgt*	(2)
pAM238-*lgt-flag*$_3$	SLP14	pSC101 origin, Plac promoter, Spc^r, expressing *E. coli lgt*	This study
pFREE		gRNA and Cas9 expressing plasmid for plasmid curing	(6)
**Primers**	**Sequence (5' -> 3')**		**Reference**
upperFLAG	CTAGAGACTACAAAGACCATGACGGTGATTATAAAGATCATGACATCGATTACAAGGATGACGATGGTACCTAGA		Proligo
lowerFLAG	AGCTTCTAGGTACCATCGTCATCCTTGTAATCGATGTCATGATCTTTATAATCACCGTCATGGTCTTTGTAGTCT		Proligo
pBAD_F	AGATTAGCGGATCCTACCTG		Sigma
pBAD_R	CTCATCCGCCAAAACAG		Sigma

References

1. Baba T, Ara T, Hasegawa M, Takai Y, Okumura Y, Baba M, Datsenko KA, Tomita M, Wanner BL, Mori H. 2006. Construction of Escherichia coli K-12 in-frame, single-gene knockout mutants: the Keio collection. Molecular Systems Biology 2.
2. Pailler J, Aucher W, Pires M, Buddelmeijer N. 2012. Phosphatidylglycerol::prolipoprotein diacylglyceryl transferase (Lgt) of Escherichia coli has seven transmembrane segments, and its essential residues are embedded in the membrane. J Bacteriol 194:2142-51.
3. Robichon C, Vidal-Ingigliardi D, Pugsley AP. 2005. Depletion of apolipoprotein N-acyltransferase causes mislocalization of outer membrane lipoproteins in *Escherichia coli*. J Biol Chem 280:974-83.
4. Diao J, Komura R, Sano T, Pantua H, Storek KM, Inaba H, Ogawa H, Noland CL, Peng Y, Gloor SL, Yan D, Kang J, Katakam AK, Volny M, Liu P, Nickerson NN, Sandoval W, Austin CD, Murray J, Rutherford ST, Reichelt M, Xu Y, Xu M, Yanagida H, Nishikawa J, Reid PC, Cunningham CN, Kapadia SB. 2021. Inhibition of Escherichia coli Lipoprotein Diacylglyceryl Transferase Is Insensitive to Resistance Caused by Deletion of Braun's Lipoprotein. J Bacteriol 203:e0014921.
5. Binet R, Wandersman C. 1995. Protein secretion by hybrid bacterial ABC-transporters: specific functions of the membrane ATPase and the membrane fusion protein. EMBO J 14:2298-2306.
6. Lauritsen I, Porse A, Sommer MOA, Norholm MHH. 2017. A versatile one-step CRISPR-Cas9 based approach to plasmid-curing. Microb Cell Fact 16:135.
